# Adaptations to extreme conditions

**DOI:** 10.7554/eLife.50647

**Published:** 2019-08-30

**Authors:** Barbara S Beltz

**Affiliations:** Neuroscience DepartmentWellesley CollegeWellesleyUnited States

**Keywords:** Rimicaris exoculata, shrimp, hydrothermal vent, brain, Other

## Abstract

The brain architecture of shrimp living in deep-sea vents provides clues to how these organisms have adapted to extreme living.

**Related research article** Machon J, Krieger J, Meth R, Zbinden M, Ravaux J, Montagné N, Chertemps T, Harzsch S. 2019. Neuroanatomy of a hydrothermal vent shrimp provides insights into the evolution of crustacean integrative brain centers. *eLife* **8**:e47550. doi: 10.7554/eLife.47550

Adaptations acquired during the course of evolution lead to changes in the form and function of organisms in response to their surroundings. For example, Darwin’s finches are famous for the amazing diversity in the sizes and shapes of their beaks, which reflects the diet of each species. But do the senses – and the associated regions of the brain – also evolve to accommodate unusual or extreme environments?

The shrimp *Rimicaris exoculata* thrives in deep-sea vents in the Mid-Atlantic Ridge, where cracks in the earth’s crust allow the boiling contents of its core to spew into the frigid depths of the ocean (Lubofsky, 2018; Figure 1). *R. exoculata* experience huge temperature swings and elevated hydrostatic pressures. Moreover, the water they live in has no oxygen and contains poisonous minerals and gases. Various studies have demonstrated anatomical and physiological adaptations to this extreme environment (Cottin et al., 2010; Ponsard et al., 2013), but adaptations of the brain and nervous system have not been explored. Now, in eLife, Steffen Harzsch of the University of Greifswald and co-workers – including Julia Machon of Sorbonne Université as first author – report how the brain of *R. exoculata* has evolved in response to low light levels and an unusual and erratic chemical environment (Machon et al., 2019).

**Figure 1.**
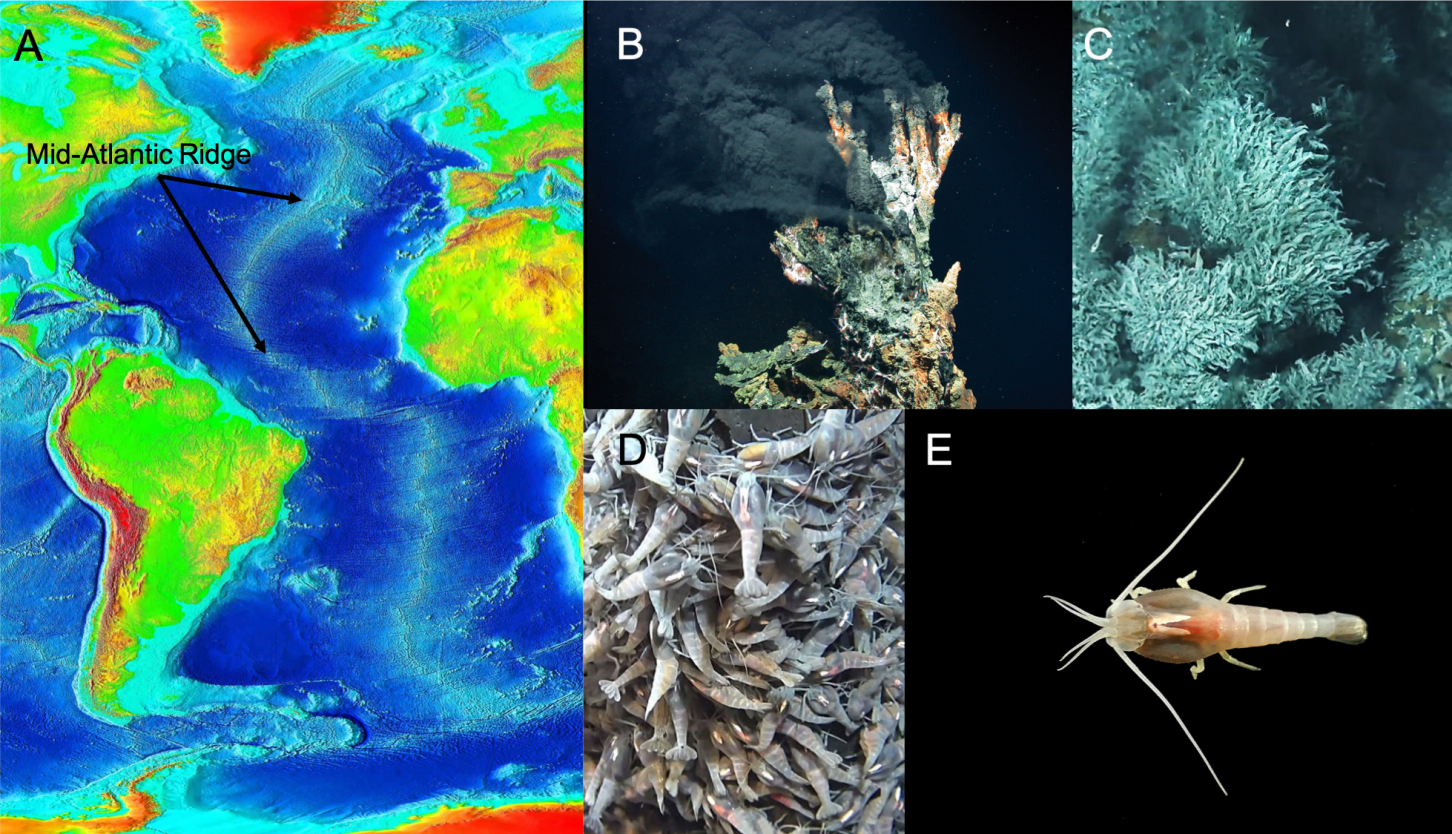
The extreme environment of *R. exoculata* has led to adaptations of its physiology and brain. (**A**) The Mid-Atlantic Ridge is the longest mountain range on Earth. It extends for about 10,000 miles and more than 90% of it is deep in the ocean. The ridge is geologically important because plates forming the earth’s crust spread apart along the Ridge, creating new ocean floor (Photograph credit: NOAA, public domain). (**B**) Hydrothermal fields along the Ridge release hot fluids from the earth’s core through chimneys, or vents, creating the environment where *R. exoculata* live (Photograph credit: MARUM − Zentrum für Marine Umweltwissenschaften, Universität Bremen - Marum, CC BY 4.0). (**C** and **D**) *R. exoculata* shrimps live along the Mid-Atlantic Ridge forming colonies with thousands of individuals that occupy the walls of hydrothermal vents (Photograph credits: IFREMER/Nautile6000, BICOSE 2018 Cruise) (**E**) Dorsal view of an individual *R. exoculata*. The shrimps have adapted to their environment, with large fixed eyes that may sense dim light and olfactory structures to detect chemicals. The architecture of the hemiellipsoid bodies in the brain is also complex, and could have a role in place memory (Photograph credit: Machon et al., CC BY 4.0).

The eyes of *R. exoculata* are large, fixed organs that do not form images but may be able to detect very dim light (Chamberlain, 2000). These shrimps also have olfactory structures on their antennae that detect dissolved chemicals, but it is unknown if or how these structures have evolved as a result of their environment. In crustaceans, the input from each sense is processed by a specific part of the brain, the architecture of which often varies depending on the functional importance of the associated sense. For example, blind crustaceans that live in caves devoid of light have reduced visual processing areas and expanded olfactory regions in the brain, presumably to increase their ability to sense their chemical environment (Stegner et al., 2015; Ramm and Scholtz, 2017). These studies suggest that mechanisms that compensate for the reduction of one sensory ability can change the architecture of the brain.

Machon et al. show that, while some blind crustaceans have partially or totally lost the areas of the brain devoted to vision, these regions are intact in *R. exoculata*. This suggests that the rudimentary eyes are light sensitive, perhaps detecting thermal radiation emitted by sea vents and various types of luminescence. Additionally, and contrary to what might be expected, there is no indication that areas involved in detecting chemicals are structurally unusual or particularly large to compensate for reduced visual abilities. This means that the visual and olfactory centers of *R. exoculata* are unremarkable and unlikely to play a dominant role in these animals.

On the other hand, a region in the brain to which these and other sensory areas connect – the hemiellipsoid body – is highly unusual. The disproportionate size and structural complexity of this region may have evolved to provide special functions that allow this animal to survive in the extreme environment of deep-sea vents. The hemiellipsoid body does not itself receive direct sensory input, but it is connected to sensory pathways in the brain, suggesting that it is involved in the integration of sensory information (McKinzie et al., 2003). However, since neither the visual nor the olfactory centers are exceptional in *R. exoculata*, the tremendous expansion of the hemiellipsoid bodies points to additional functions for this region beyond sensory integration.

Experimental evidence in other crustaceans indicates that the hemiellipsoid bodies may play a role in memory (Maza et al., 2016). Machon et al. suggest a specific role in place memory, the type of memory used to associate a specific place with events or objects. Place memories are formed when animals find their way to specific locations. For example, in humans, they allow us to remember where we were at a given time. This place memory hypothesis for the hemiellipsoid bodies of *R. exoculata* is based on comparisons with insects and with other crustaceans that migrate long distances (Wolff et al., 2017; Krieger et al., 2012). Place memory may provide essential navigational skills for *R. exoculata*, because remembering the locations of hot vent chimneys and boiling emissions is critical for survival.

The observations of Machon et al. provide an intriguing window into evolutionary changes in the brain that may be directly related to the success of *R. exoculata* in deep-sea vents. Further experiments should test the functional roles of the hemiellipsoid bodies in crustaceans, and how this part of the brain contributes to the processing of sensory information and to memory.
